# Protein tyrosine phosphatase PTPN22 +1858C/T polymorphism is associated with active vitiligo

**DOI:** 10.3892/etm.2014.1975

**Published:** 2014-09-17

**Authors:** MARTHA ELENA GARCIA-MELENDEZ, MAURICIO SALINAS-SANTANDER, CELIA SANCHEZ-DOMINGUEZ, HUGO GONZALEZ-CARDENAS, RICARDO M. CERDA-FLORES, JORGE OCAMPO-CANDIANI, ROCÍO ORTIZ-LÓPEZ

**Affiliations:** 1 Dermatology Service, Hospital Universitario ‘Dr. José Eleuterio González’, Monterrey, CP 64460, Nuevo León, Mexico; 2 Department of Biochemistry and Molecular Medicine, Faculty of Medicine, Universidad Autónoma de Nuevo León, Monterrey, CP 64460, Nuevo León, Mexico; 3 Saltillo Unit Faculty of Medicine, Universidad Autónoma de Coahuila, Saltillo CP 25000, Coahuila, Mexico; 4 Nursery School Faculty, Universidad Autónoma de Nuevo León, Monterrey, CP 64460, Nuevo León, Mexico; 5 Molecular Biology, Genomics and Sequencing Unit, Center for Research and Development in the Health Sciences, Universidad Autónoma de Nuevo León, Monterrey, CP 64460, Nuevo León, Mexico

**Keywords:** vitiligo, PTPN22 +1858C/T, lymphoid protein tyrosine phosphatase, autoimmune diseases, polymerase chain reaction-restriction fragment length polymorphism, Mexican population, polymorphism

## Abstract

Vitiligo is characterized by a skin depigmentation disorder resulting from an autoimmune response targeting melanocytes. Within the genetic factors involved in the development of the vitiligo immune response, various genes in the major histocompatibility complex (MHC) and non-MHC loci have been considered to be risk factors. The *PTPN22* gene encodes for a lymphoid protein tyrosine phosphatase, a regulator of the activation and development of T-cells. The *+1858C/T* polymorphism has been associated to autoimmune disease susceptibility in different populations and could be implicated in the onset of vitiligo. To assess the possible association between the presence of *PTPN22 +1858C/T* and vitiligo, 187 patients with vitiligo and 223 control subjects were analyzed in the study. Genomic DNA was isolated using the salting-out method and samples were subjected to polymerase chain reaction-restriction fragment length polymorphism in order to detect the *PTPN22 +1858C/T* polymorphism. Causal associations were determined by χ^2^ test and their respective odds ratio (OR) was assessed in a 2×2 contingency table. The results showed an association between active vitiligo and the allele T load [P=0.0418; OR, 2.5706; 95% confidence interval (CI), 1.0040–6.5816], and active vitiligo-CT genotype (P=0.0389, OR, 2.6548; 95% CI, 1.0191–6.9156). In conclusion, the present data indicates a possible association between the *PTPN22 +1858C/T* genotype and a significant susceptibility of developing an active form of vitiligo.

## Introduction

Vitiligo is an acquired multifactorial progressive depigmentation disorder, characterized by the presence of circumscribed white macules on the skin in which the progressive loss of functional melanocytes in the epidermis is observed (1,2). Vitiligo has been reported to affect 0.1–2% of the population worldwide, but the prevalence varies considerably among the populations or ethnic groups analyzed (1,3,4) and can be as high as those reported in Mexico (4%) or India (8.8%), affecting the two genders equally (5). Furthermore, vitiligo is considered an autoimmune disease (AID), as it can be associated with AIDs, including Hashimoto’s thyroiditis, diabetes mellitus, Addison’s disease, alopecia areata and ophthalmic abnormalities, such as iritis (4,6–8). This association is supported by: i) The presence of lymphocytes in the dermis of early lesions, ii) antibodies against melanocytes and antigens from the melanogenic system in a number of patients with active disease (9,10), and iii) the response to treatment with immunomodulatory agents, including corticosteroids and phototherapy (11,12).

Autoimmunity is responsible for the misdirected attacks by the immune system of the body against itself, as a result of the failure to recognize self-antigens correctly. This phenomenon is usually a harmless process, but has the potential to lead to a broad spectrum of multiple complex AIDs. Genetic and environmental factors are believed to be the triggers of progression from harmless autoimmunity to AID. The major histocompatibility complex (MHC) locus appears to be a major genetic factor that confers susceptibility to multiple AIDs (13). However, a non-MHC gene may contribute to risk in different AIDs (12).

The *PTPN22* gene located on chromosome 1p13.2 encodes a lymphoid protein tyrosine phosphatase (LYP) (14) that is important as a negative control of T-cell activation and T-cell development. PTPN22 belongs to a family of protein tyrosine phosphatases that are involved in the prevention of spontaneous T-cell activation by dephosphorylation and inactivation of T-cell receptor-associated kinases and their substrates (15). PTPN22 is specifically expressed in lymphocytes and suppresses the downstream mediators of T-cell receptor signaling through binding with C-terminal Src kinase (CSK) (15).

Polymorphic variants of this gene have been previously associated with various AIDs. The *+1858C/T* single-nucleotide polymorphism (SNP) (rs2476601), in exon 14 of the *PTPN22* gene has been associated with susceptibility to several diseases and dermatological pathologies in different populations (15–18). This polymorphism results in an amino acid change from arginine (Arg) to tryptophan (Trp) in codon 620. The functional significance of this SNP has been discussed by a number of studies (19–22). Experimental evidence indicates that the disease-associated LYP variant Trp620 prevents the interaction of LYP with CSK (19). As a result, the T-cell receptor-associated kinases may be able to induce T-cell activation in an uncontrolled manner and this may increase the overall immune system reactivity and predispose an individual to AID (20,21). Other studies (21–23) have demonstrated that the *+1858T* allele is a hypomorphic variant, which means that the polymorphic variant of the protein has a diminished function and cannot inhibit the T cell receptor signaling in T cells correctly; therefore, LYP-620Trp is considered a loss-of-function protein. This impaired LYP function in T cells may drive hyperresponsive B cells to break the tolerance in the periphery and to the production of autoantibodies, leading to the development of AID (22).

In vitiligo, the SNP rs2476601 has been identified as a risk factor for a specific generalized form in European Caucasian populations of Romania (23). The data collected for this gene in Mexico are obsolete or limited. The results obtained by different studies support the association of the *PTPN22 1858T* allele with sporadic childhood-onset systemic lupus erythematosus (SLE) in the Mexican population (24), and susceptibility to rheumatoid arthritis in the Western Mexican population (16). However, thus far, no similar studies have been carried out on vitiligo. The aim of the present hospital case-control study was to investigate the possible involvement of the *PTPN22* gene *1858C/T* polymorphism in Mexican patients with vitiligo from Northeast Mexico.

## Materials and methods

### Selection of subject and controls

A total of 187 Mexican patients diagnosed with vitiligo and recruited by the Dermatology Department of the ‘Dr Jose E. Gonzalez’ University Hospital of the Universidad Autonoma de Nuevo León (UANL; Monterrey, Mexico), were selected in the study conducted between November 2009 and May 2010 (from the states of Coahuila, Nuevo León, San Luis Potosi, Tamaulipas and Zacatecas). The University Hospital-UANL Institutional Review Board approved and registered the study under the code DE08-008. Subsequent to providing their informed consent, the patients were evaluated and surveyed via direct interview.

The information gathered from the patients included: Age, gender, birthplace, full clinical history, age of disease onset, previous treatment (if any), vitiligo lesions in previously traumatized regions (Koebner’s phenomenon), class and distribution of vitiligo in the patient and their families. The patients were further classified as exhibiting the following types of vitiligo: Focal (one or more maculae in an unsegmented pattern); segmented (one or more maculae distributed in a single dermatome or zosteriform pattern), vulgaris (symmetric or asymmetric distribution of maculae in one or more areas) and universal (>80% skin depigmentation). The course of the disease was classified as active (progressive, appearance of new maculae in the last six months) or stable.

The control group consisted of 232 patients from the Mexican Mestizo population who were recruited during an earlier study from a general unbiased healthy population through the Blood Bank of the aforementioned hospital. For study purposes, a Mexican Mestizo was defined as a person born in Mexico, whose last three ascending generations were also born in the country.

### Experimental procedure

#### DNA isolation

Peripheral venous blood samples were obtained from the patients with vitiligo and the controls. The samples were centrifuged, and the buffy-coat was processed for DNA isolation by the salting-out method and finally suspended in Tris-EDTA (pH 7.8), at a final concentration of 0.1–1.0 μg/μl.

#### PTPN22 +1858C/T genotyping

Genotyping was performed by polymerase chain reaction-restriction fragment length polymorphism (PCR-RFLP) as modified by Harrison *et al* (25). PCR was carried out using an MJ Research PTC 100 thermal cycler (Bio-Rad, Hercules, CA, USA) as follows: A total amount of 250 ng genomic DNA, 0.5 μM *PTPN22 +1858C/T* primers (forward, 5′-ATGTTGCTTCAACGGAATTT-3′ and reverse, 5′-CATGCTGCTATTGCTCTGCT-3′), 0.2 mM deoxyribonucleotide triphosphates, 1.5 mM MgCl_2_ and 2.5 units of Taq DNA polymerase (Promega Corporation, Madison, WI, USA) at 94°C/1 min, 58°C/1 min and 72°C/1 min (33 cycles). The PCR samples (~1 μg) were subsequently digested overnight with *Xcm*I (New England Biolabs, Ipswich, MA, USA) at 37°C. The digested amplicons were analyzed by electrophoresis in 2% agarose gels using ethidium bromide.

#### Statistical analysis

The collected data were organized in a database and statistical calculations were performed with the SPSS 17.0 software for windows (SPSS, Inc. Chicago, IL, USA) and the Epi-INFO^™^ 7 statistical program (USD Incorporated 2008, Stone Mountain, GA, USA). The Hardy-Weinberg equilibrium was obtained using a goodness-of-fit test. Genotypic dependence between the patients and controls was determined by the χ^2^ test, and the odds ratio (OR) was calculated from 2×2 contingency tables. The Mann-Whitney U test and Spearman’s Rho were used to determine the correlation of age, gender and genotype for each studied group. P≤0.05 was considered to indicate a statistically significant difference.

## Results

### Clinical characteristics of the participants

The present study analyzed the genotype distribution and allele frequency for *PTPN22 +1858C*/*T* polymorphism corresponding to codon 620 (620Arg-Trp). The main clinical features of the participants are summarized in Table I.

The study consisted of 187 patients with vitiligo, including 97 (51.9%) females and 90 (48.1%) males. The medium age of the patients at the time of consultation was 41.33±16.194 years, with a median of 44 years. The mean age of vitiligo onset was 26.28±15.752 years, with a median age of 10 years. The patients with vitiligo were classified into four categories according to the type of vitiligo, and into two main groups depending on disease activity (Table I). The appearance of skin lesions on lines of trauma or Koëbner phenomenon and common comorbidities, usually described as AIDs, are shown in Tables I and II.

The most common illness associated with vitiligo was thyroid alterations, in which hypothyroidism was present in 19 patients (10.2%), subclinical hypothyroidism in 12 patients (6.4%), hyperthyroidism in five patients (2.7%) and subclinical hyperthyroidism in only one patient (0.5%). In order to assess the Hardy-Weinberg equilibrium, the data were analyzed by means of a χ^2^ goodness-of-fit test. Several tests were used (control group, Pearson test P=0.763198, likelihood-ratio (Llr) test P=0.673069, Fisher’s exact test P=1.000000; case group, Pearson test P=0. 541091, Llr test P=0.397683, Fisher’s exact test P=1.000000), all of which had a P-value >0.05, indicating that the rs2476601 genotype was in Hardy-Weinberg equilibrium in patients with vitiligo and the controls. The statistical results obtained from the study are shown in Table III.

### Association of the genotypes in patients with vitiligo

When the PTPN22 +1858C/T genotypes and allele frequencies were compared between the whole cohort of cases and controls, evidence of association was observed (Table III). The CT genotype was more frequent in patients with vitiligo than in controls (P=0.0445). However, no significant association was observed in the T allele of patients with vitiligo when comparing all the vitiligo types against controls.

Finally, when considering population variables, including gender, type of vitiligo, age of disease onset, immune pathologies and disease activity, an association was only observed between the active vitiligo-allele T load (P=0.0418; OR, 2.5706; 95% confidence interval (CI), 1.0040–6.5816) and active vitiligo-CT genotype (P=0.0389; OR, 2.6548; 95% CI, 1.0191–6.9156) (Table III).

## Discussion

Vitiligo is a complex depigmentation disorder in which there is progressive and selective destruction of the cells that produce the pigment of the skin, hair and eyes, known as melanocytes (26). Vitiligo is believed to have a non-Mendelian genetic predisposition pattern, with a polygenic and multifactorial inheritance (27). Multiple trigger factors have been associated with the development of vitiligo, including stress, infections, traumatisms, malignancy, sun exposure, neurological abnormalities, and endocrine and autoimmune alterations, which can act independently or in combination (28).

Previous studies associate vitiligo to autoimmune pathologies due to its frequent association with diseases of this nature, in which melanocyte cell death by immune-mediated apoptosis is considered an important susceptibility factor for vitiligo, emphasized by the detection in serum of auto-antibodies and reactive T lymphocytes targeted against this cell type (29). In a number of case-control genetic studies of vitiligo, a genetic association of the MHC was observed by testing different MHC markers in patients with different vitiligo phenotypes versus controls (30). Since MHC loci have been associated with the development of vitiligo, identification of non-associated MHC loci with this pathology and immune diseases have been reported. The *PTPN22* locus is one of the strongest risk factors outside of the MHC that associates with AIDs (31).

The product of the *PTPN22* gene is a phosphatase known as LYP, which acts as a negative regulator of T cell signaling. Since LYP is also expressed in other cell types, such as B cells, monocytes, neutrophils and dendritic cells (16), the presence of the +1858T allele results in a loss of functional proteins that are unable to regulate T cell activation leading to a hyperresponsive phenotype of T, B and dendritic cells (16,32), thereby having a possible role in the pathogenesis of vitiligo. This observation is supported by a previous study that described the increase of Th17 and activated dendritic cells and cellular immune elements in the active margins of vitiligo lesions, which could potentially drive the inflammatory responses in the skin (33).

In the current study, the presence of the *PTPN22 +1858C/T* polymorphism in a population affected by vitiligo from northeast Mexico was analyzed. Even though the *PTPN22 +1858C/T* polymorphism has been reported in Mexico for other diseases, the data remain limited. Previous studies in Mexican populations have described the allele and haplotype frequency distributions of the *PTPN22* gene (34) in SLE (24) and susceptibility to rheumatoid arthritis (15).

In the case of vitiligo, there are few studies worldwide that make reference to the development of this pathology and its association with this polymorphism. There is evidence that the *PTPN22 +1858T* allele constitutes a generalized risk for vitiligo in a European Caucasian (Romanian) population, highlighting the importance of a genetically mediated autoimmune mechanism in the pathogenesis of vitiligo (23). By contrast, studies of the segmental form of the disease in Egyptian females failed to find an association with the presence of this polymorphism (35). Some of these positive findings have been confirmed by a meta-analysis performed on European and Asian populations by Song *et al* (36) in 2013. The *PTPN22 +1858C/T* polymorphism has not been previously assessed for vitiligo in American populations. Although an association was found between all the vitiligo types analyzed in general and the studied polymorphism (P=0.0445; OR, 2.3184; 95% CI, 1.0003–5.3736) this result was not sustainable when focusing on the T allele (P=0.0479; OR, 2.2595; 95% CI, 0.9869–5.1730), however, a notable association was found for the active forms of vitiligo and the T allele load of the polymorphism (P=0.0389), which conferred an increased OR of 2.6548.

In conclusion, the present data indicate the involvement of the *PTPN22 +1858T* allele as a genetic risk factor for susceptibility to active vitiligo in the Mexican population, supporting the evidence of the possible involvement of the immune response in the pathogenesis of vitiligo.

## Figures and Tables

**Table I:** General characteristics of patients with vitiligo.

Type of vitiligo	Gender, n (%)		Vitiligo activity, n (%)		Koëbner phenomenon, n (%)	Age of onset		
		
	Female	Male	Active	Stable		Age (years)^a^	<30 years, n (%)	≥30 years, n (%)
Vulgaris	88 (47.06)	78 (41.71)	85 (45.45)	81 (43.32)	47 (25.13)	26.46±15.201	93 (39.04)	73 (35.35)
Universal	1 (0.53)	2 (1.07)	-	3 (1.6)	-	12.33±15.373	2 (1.07)	1 (0.53)
Focal	7 (3.74)	10 (5.35)	6 (3.21)	11 (5.88)	2 (1.07)	28.41±19.912	9 (4.81)	8 (4.28)
Segmental	1 (0.53)	-	1 (0.53)	-	-	3	1 (0.53)	-

a Age is presented as the mean ± standard deviation.

**Table II:** Autoimmune pathologies associated with vitiligo in patients.

	Autoimmune pathologies								
	
Type of vitiligo	Thyroid	AA	DM	HTN	Atopy	SLE	RA	MG	AH
VV, n (%)	33 (17.65)	5 (2.67)	13 (6.95)	23 (12.3)	6 (3.21)	1 (0.53)	1 (0.53)	1 (0.53)	1 (0.53)
UV, n (%)	1 (0.50)	-	-	-	-	1 (0.53)	-	-	-
FV, n (%)	3 (1.52)	1 (0.53)	1 (0.53)	1 (0.53)	1 (0.53)	-	-	-	-
SV, n (%)	-	-	-	-	1 (0.53)	-	-	-	-
Total, %	19.78	3.21	7.49	12.83	4.29	1.07	0.53	0.53	0.53

AA, alopecia areata; DM, diabetes mellitus; HTN, arterial hypertension; SLE, systemic lupus erythematosus; RA, rheumatoid arthritis; MG, myasthenia gravis; AH, autoimmune hepatitis; VV, vulgaris vitiligo; UV, universal vitiligo; FV, focal vitiligo; SV, segmental vitiligo.

**Table III:** Frequencies of PTPN22 +1858C/T genotypes and alleles in patients with vitiligo and healthy control subjects.

Genotypes and alleles	Patients with vitiligo, n (%)	Controls, n (%)	χ^2^	OR	95% CI	P-value
All vitiligo types						
Genotypes						
CC	171 (91.44)	223 (96.12)	4.0366	0.4313	0.186–10.9997	0.0445
CT	16 (8.56)	9 (3.88)	4.0366	2.3184	1.0003–5.3736	0.0445
TT	0 (0)	0 (0)				
Alleles						
C	358 (95.72)	455 (98.06)	3.9124	0.4426	0.1933–1.0133	0.0479
T	16 (4.28)	9 (1.94)	3.9124	2.2595	0.9869–5.1730	0.0479
Active vitiligo						
Genotypes						
CC	84 (90.32)	223 (96.12)	4.266	0.3767	0.1446–0.9812	0.0389
CT	9 (9.68)	9 (3.88)	4.266	2.6548	1.0191–6.9156	0.0389
TT	0 (0)	0 (0)				
Alleles						
C	177 (95.16)	455 (98.06)	4.1445	0.389	0.1519–0.9960	0.0418
T	9 (4.84)	9 (1.94)	4.1445	2.5706	1.0040–6.5816	0.0418
Stable vitiligo						
Genotypes						
CC	88 (92.63)	223 (96.12)	1.7632	0.5074	0.1833–1.4044	0.1842
CT	7 (7.37)	9 (3.88)	1.7632	1.971	0.7121–5.4555	0.1842
TT	0 (0)	0 (0)				
Alleles						
C	183 (96.32)	455 (98.06)	1.719	0.5171	0.1898–1.4092	0.1898
T	7 (3.68)	9 (1.94)	1.719	1.9338	0.7096–5.2700	0.1898

OR, odds ratio; CI, confidence interval.
